# A Precision Driver Device for Intraoperative Stimulation of a Bone Conduction Implant

**DOI:** 10.1038/s41598-020-58512-7

**Published:** 2020-02-04

**Authors:** Mohammad Ghoncheh, Thomas Lenarz, Hannes Maier

**Affiliations:** 1 Cluster of Excellence Hearing4all, Hannover, Germany; 2 Department of Otolaryngology and Institute of Audioneurotechnology (VIANNA), Hannover Medical School, Hannover, Germany

**Keywords:** Biomedical engineering, Biophysics, Medical research

## Abstract

Semi-implantable bone conduction implants (BCI) and active middle ear implants (AMEI) for patients with sensorineural, conductive or mixed hearing loss commonly use an amplitude modulation technology to transmit power and sound signals from an external audio processor to the implant. In patients, the distance dependence of the signal amplitude is of minor importance as the skin thickness is constant and only varies between 3–7 mm. In this range, critical coupling transmission technique sufficiently reduces the variability in amplitude, but fails to provide well-defined amplitudes in many research and clinical applications such as intraoperative integrity tests where the distance range is exceeded by using sterile covers. Here we used the BCI Bonebridge (BB, Med-El, Austria) as an example to develop and demonstrate a system that synthesizes the transmission signal, determines the distance between the transmitter and the receiver implant coil and compensates transmission losses. When compared to an external audio processor (AP304) on an artificial mastoid, our system mainly decreased amplitude variability from over 11 dB to less than 3 dB for audio frequencies (0.1–10 kHz) at distances up to 15 mm, making it adequate for intraoperative and audiometric tests.

## Introduction

**Figure 1:**
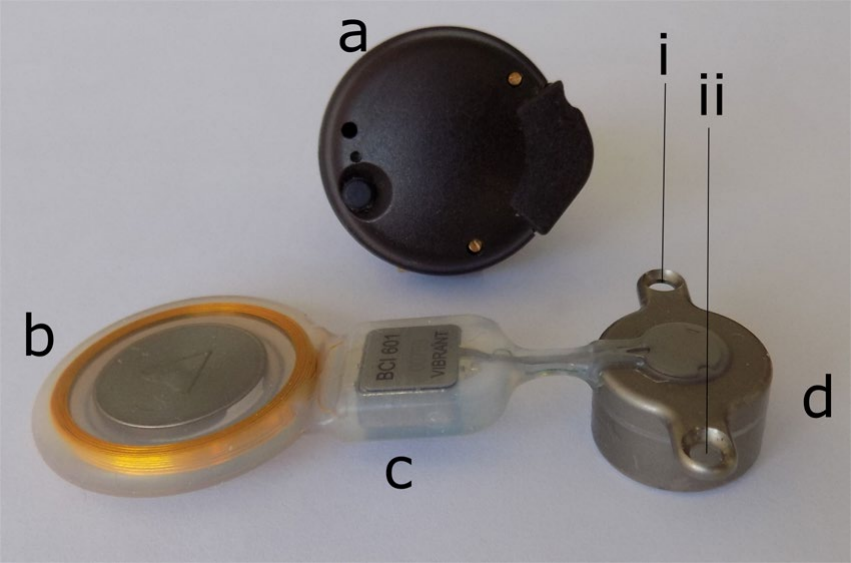
The Bonebridge consists of two parts: (**a**) an external audio processor containing microphone, battery, signal processing and the transmitter coil and the implant with a (**b**) receiver coil, (**c**) a demodulator and (**d**) a bone conduction floating mass transducer (BC-FMT) to be coupled to the mastoid bone by two screws (i, ii). Source: Original.

**Figure 2:**
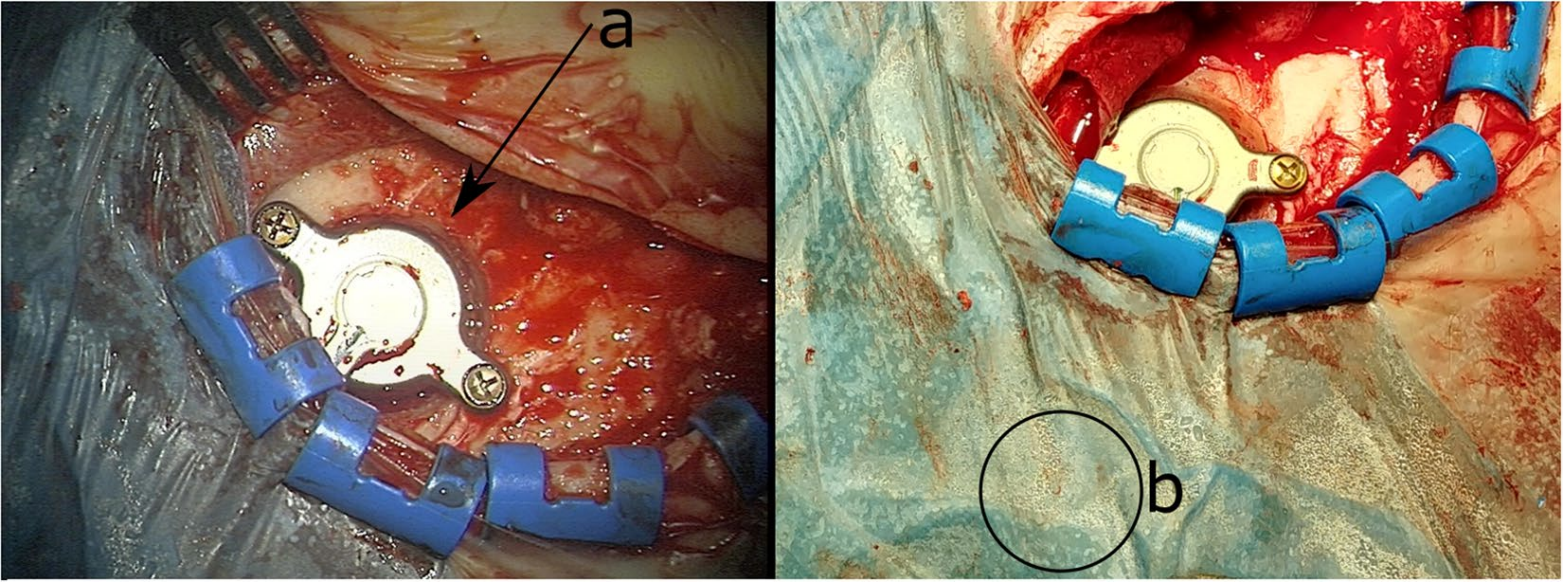
Typical surgical view of a BB implantation illustrating (**a**) how the BC-FMT is firmly attached to the mastoid bone and (**b**) where the transmitter coil is placed on the sterile cover driving the BB. Source: Photos were originally taken at our clinic.

The Bonebridge (Fig. 1, BB, MED-EL, Innsbruck, Austria) transcutaneous bone conduction implant (TBCI), recently certified and introduced to the market, is used to treat patients with conductive or mixed hearing loss^1^. The BB is a semi-implantable device that consists of two separate parts: an audio processor (Fig. 1a) that records sound via microphones and transmits it wirelessly with an analogue amplitude modulation (AM) radio frequency (RF) link to the subcutaneous implant. The receiver coil in the implant picks up the signal (Fig. 1b), which is then demodulated (Fig. 1c), driving the bone conduction floating mass transducer (Fig. 1d, BC-FMT) that is firmly attached to the mastoid by two screws (see Figs. 1d(i,ii) and 2a). Here, the demodulated audio signal stimulates the cochlea with bone vibration, thus bypassing the middle ear^2^. For quantitative measurements such as those performed intraoperatively to verify the functionality of the implant or audiological testing, transmitting a well-characterized stimulus amplitude to the implant is required. The implant’s external processor transmission system is designed for daily use in patients with normal skin thickness of approximately 5 to 7 mm. This distance between the audio processor and the implant should be kept less than 7 mm^3^. The “critical coupling” technique ensures a constant transmission in this range^4^^,^ and the audio processor fitting and output level are not affected since the individual skin thickness remains constant. However, using the audio processor in operation theatres as a way to transmit the stimuli to the implant is different in terms of the distance between the transmitter and the receiver coil. Here the gap could be up to 15 mm due to the use of sterile covers on patients (Fig. 2b), and this distance is unknown and often unclear if the coils are geometrically aligned. Under these specific circumstances this extended distance range plays an important role in the analogue transmission as it introduces inaccuracies due to transmission losses at distances larger than the optimized range^5^.

As a consequence of the larger and variable distance, a stimulus of uncalibrated amplitude is being transmitted to the implant. Therefore, the interpretation of the input signal and the measured response is unreliable and causes an undesired inter-individual variability in any collected data. To transmit a well-defined stimulus, it was necessary to develop a transmission and measurement system that allows for output level compensation of the vibrator transducer output by estimating the unknown distance between the transmitter and the receiver implant coil as well as to compensate for attenuations. The aim of this study was to develop a device to transmit well-defined stimuli to TBCIs in order to use it for research purposes. Therefore, we designed a transmission system that is able to drive amplitude modulation transmission-based implants with a calibrated input. The system first determines the lateral distance between the transmitter and receiver coil by measuring the electrical properties of the transmission coil. Then the input signal amplitude is adjusted to compensate for transmission losses arising from the distance, increasing the accuracy.

## Methods

In this study, we developed an RF link to transmit a calibrated stimulus signal to the TBCI. AM signals were synthesized and measurements were performed using a multifunctional data acquisition device (DAQ, NI-6356 BNC, National Instruments, USA) with a 16-bit ADC (analogue-to-digital converter) resolution in combination with a custom-written program in LabVIEW 2015 (National Instruments, USA). A BB implant was used to compare the transmission properties of our transmission system in terms of output dependency to the distance between the transmitter and receiver coil with a modified older generation AP 304 audio processor (MED-EL, Innsbruck, Austria) in which the modulator is separate and can be connected electrically, thus circumventing the processor. For testing purposes, an artificial mastoid (type 4930, B&K, Denmark) was used to measure and compare the output acceleration of the BC transducer driven with both driver systems. With the TBCI on the artificial mastoid, the output vibration level of the implant driven with the AP 304 was compared to our developed transmission system with varying distances ranging from 0–15 mm between the respective transmitter and the implant coil housings. This covers the potential distance range commonly seen during implantation surgeries in our clinic. Additionally, the total harmonic distortion (THD) was computed using the first five available harmonics of each stimulus frequency. The data were sampled at 302 kS/s in all measurements and averaged at least 50 times to obtain a SNR (signal-to-noise ratio) above 12 dB. To fulfil the safety requirement regarding the electrical insulation from the powerline similar to IEC 60601–1, the DAQ was powered with an isolated power supply which was approved for medical device applications (EN/IEC60601–1, KMS40–12, TDK-Lambda Corporation). The transmission circuit was powered by batteries and the controlling laptop was disconnected from power during measurements.

### Signal generation

#### Our system

To drive our transmission system, the carrier of the synthesized AM signal was a 151 kHz triangular signal generated by switching between −*A*_*c*_ and + *A*_*c*_ at the sample rate. The modulation signals were sine waves with frequencies ranging from 0.125 to 10 kHz (147.5, 258.1, 516.3, 737.6, 995.7, 1512.0, 1991.5, 2987.2, 4019.8, 5015.6, 6011.3, 8002.8, 9994.3 Hz) that were closest to audiometric frequencies with respect to our frequency resolution (F_s_ = 36.87 Hz). The modulation signal was fitted to 8192 sample frames to generate a pseudo-periodic signal and to avoid the necessity for windowing during the FFT computations. The frames were repeated and averaged to obtain a signal-to-noise ratio above 12 dB in all measurements. The same 13 frequencies mentioned above were used in all the recordings where the vibration output of the TBCI was measured. Filtering of the carrier was not performed and removal of higher harmonics components of the carrier was left to the low-pass characteristics of the implant circuit. The modulated wave can be described by the Eq. 1:

1 $${v}_{in}({t}_{n})={A}_{c}(1+m\,sin(2\pi {f}_{m}{t}_{n}))S({t}_{n})$$

where A_c_, m and f_m_ are the amplitude of the carrier, the modulation index (m) and the modulating frequency, respectively. Time at each sample (n), represented by t_n_ and S(t_n_) is defined as:

$$S({t}_{n})=\{\begin{array}{c}-1\,\,\,\,\,\,2n\\ +1\,\,\,\,\,\,2n-1\end{array}\,\,for\,n=(1,2,\ldots ,8192);\,{t}_{n}=(n-1)/{F}_{s}$$

Stimulation strategy. To minimize distortions and to take the maximal output range of the buffer amplifier into account we divided our stimulation strategy into three domains for the transmitted input signal amplitude. (1) Below a modulation signal amplitude of 0.2 V_p_ the carrier amplitude A_c_ was kept constant at 1 V_p_ and the modulation index was increased up to a limit of 20% to enhance the output vibration level. (2) For modulation signal amplitudes between 0.2 and 0.8 V_p_, the modulation index was kept constant at 20% and the carrier voltage was increased up to 4 V_p_. (3) Above that, A_c_ was kept constant at 4 V_p_ and the modulation index was increased to a maximum of m = 50%, resulting in a maximum peak amplitude of ± 6 V_p_ at the output of the buffer amplifier that was powered by ± 9 V batteries. This scheme was chosen to avoid non-linear behaviour of the demodulator and output amplifier and was used in all experiments shown here.

#### AP 304

The stimuli to drive the AP 304 were the 13-sine waves from 0.1 to 10 kHz with input amplitude of 0.1 V_p_ to the internal modulator of the external processor. Synthesized signals were generated using the analogue output of the DAQ using the same above-mentioned sample frequencies and frames.

### Inductive link

**Figure 3:**
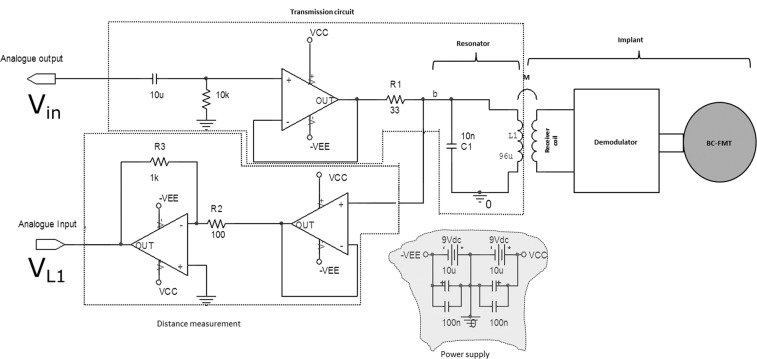
Schematic of the transmission circuit diagram. The separate transmitter coil L_1_ is connected to the transmission circuit by an exchangeable 3-meter RG174 coaxial cable with SMA connectors (see nod b).

The main principle of the inductive link signal transmission with AM technique has been extensively explained in previous publications^6–8^. This transmission system works based on two mutually coupled RF coils that form a transformer. The AM signal of the DAQ was buffered with a low-noise operational amplifier to drive the original transmission coil (courtesy of MED-EL, Innsbruck, Austria) of the sound processor. The amplifier circuit was powered symmetrically with 9 V batteries. The resonator was tuned to the carrier frequency of 151 kHz using a 10 nF capacitor (C1 in Fig. 3). The transmission coil of the external audio processor is an air core inductor with an inner diameter of 2.3 cm and an inductance of 96 µH. The transmitter coil was encapsulated in a small thermoplastic polymer (ABS) housing and connected to the amplifier with a 3-meter long RG174 coaxial cable.

### Evaluation of the transmission system

**Figure 4:**
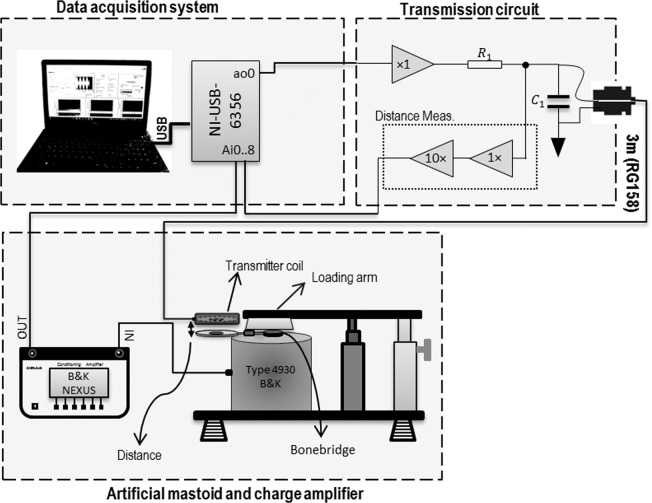
Measurement setup and block diagram of the transmission system and implant.

For comparison to the system we developed, the modified AP 304 was used to drive the implant. For both the AP 304 and our system, the acceleration output of the implant was measured by placing the BC transducer on the artificial mastoid. The measurement setup is shown in Fig. 4. The loading arm of the artificial mastoid was adjusted to apply 5.4 N static force to press the vibrator body against the artificial mastoid surface. During implantations, the body of the BC transducer is firmly screwed to the human mastoid and may provide a different sound transmission characteristic to the skull compared to the measured output of BC transducer on the artificial mastoid. However, this configuration at a well-defined force allowed for a relative comparison of acceleration/force output to the skull although the absolute output may depend on the specific fixation. The acceleration output was amplified (100 mV/ms^−^²) using a conditioning amplifier (NEXUS, type 2692, B&K, Denmark) and was recorded using the analogue input of the DAQ with the same sample rate of 302 kS/s and 16-bit ADC resolution.

The thickness of the AP 304 housing adds approximately 1 mm to the coil–coil distance and the housing of our system approximately 2.3 mm. All distances in the following measurements correspond to the distances between the outer surfaces, disregarding the additional thicknesses of the housings and the silicone cover of the implant. The distance between the respective driver system (this study vs. AP 304) and the implant receiver coil was varied by placing 1-mm PVC spacers between the coils. Although dielectric properties of tissue may have a significant effect on the relative permittivity ε, namely the α- and β-dispersion at low frequencies^9^ the used Helmholtz-similar configuration of coils here depends mainly on the magnetic field. As the relative magnetic permeability µ of most tissues is close to that of free space in the frequency range used it can be expected to be of minor importance and a polymer spacer can be assumed a good equivalent. However, this needs not to be true for the used magnets to hold the coils in place, but their effect is identical in the test and application and already included. The acceleration amplitude and phases were measured when the distance between the AP 304 and the implant receiver coil was altered from 0 mm to 15 mm. At 0 mm, the transmitter coil/AP 304 housings were directly placed on top of the receiver coil (BB implant) without any spacers, and different numbers of 1-mm spacers were placed at all other distances between the coils.

For comparison, we set the stimulus level of the AM signal in our transmission system to 1.5 V_p_ (*A*_*c*_) with a modulation index of m = 20% to obtain approximately similar levels of acceleration amplitude as with the AP 304 at 100 mV_p_ input.

### Distance compensation

As the amplitude acceleration level changes due to distance transmission losses, we measured the amplitude voltage across L_1_ that is accessible in the driver circuit. These values were used to determine the coil–coil distance and to indicate the amount of expected transmission loss. The amplitude changes due to distances were then compensated with the adaption of input to the transmission circuit.

#### Amplitude changes

The vibration amplitude of the BC transducer driven with the AP 304 at 6 mm provided the maximum vibration amplitude, presumably because the processor is optimized for patients’ normal skin thickness (5 and 7 mm). Similar output vibration level amplitude of the BC transducer was achieved with our transmission device when the input signal to our transmission circuit was (A_c_ = 1.5 V_p_/m = 20%). Using this electrical input to our transmission system, decrease of the acceleration amplitudes due to distance variation between the coils was measured on the artificial mastoid.

#### Determination of the coil–coil distance

**Figure 5:**
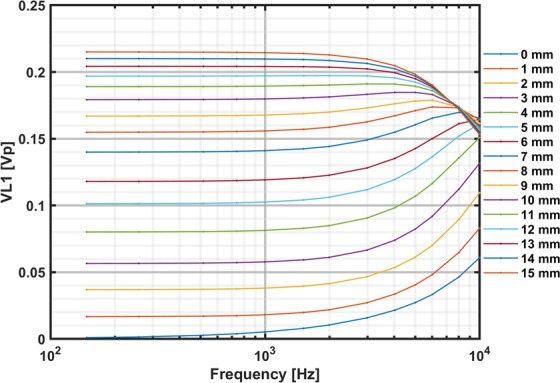
The voltages across the primary coil (V_L1_) at distances from 0 to 15 mm between 0.1 and 10 kHz. The carrier voltage was A_c_ = 0.2 V_p_ and m = 20%. The results V_L1_ shown here are after 10-fold amplification.

In an inductive link, the mutual inductance between the transmitter and receiver coil is proportional to the coupling coefficient that is related to the distance between the coils and the self-inductance of each coil^7^. The voltage across L_1_ (Fig. 3) in the primary circuit due to the coupling coefficient changes with distance was used to estimate the distance between the transmitter and receiver coil. The amplitude voltage across L_1_ was buffered, amplified 10 times and was recorded using the analogue input of the DAQ to determine the distance between the coils. Distance variations from 0 mm to 15 mm were achieved by placing a 1-mm spacer between the transmitter and receiver coil. To obtain a distance estimate from V_L1_ with optimum resolution without driving the transmission circuit into saturation for distance measurements, the carrier amplitude was kept at A_c_ = 0.2 *V*(m = 20%) between 0.1 and 10 kHz. With this procedure, the frequency-specific distance - V_L1_ relationship was determined using the lower sideband of the FFT to determine the voltage V_L1_ for each specific distance (see Fig. 5). As the measured voltage was mainly independent at frequencies below 1 kHz, we chose 1 kHz to determine V_L1_ values to estimate the distance. The low amplitude of *A*_*C*_ also has the advantage that the system can be used in awake patients leading to an output well below maximum level. The average amplitude of the lower sideband of the modulation signal (V_L1_) across 10 repeated measurements and the min-max boundaries at each distance were used as the reference value to estimate the distance and its accuracy in an intraoperative scenario where measured V_L1_ will correspond to a certain distance (see Table 1).

**Table 1:** Average voltage measured across L_1_ at 1 kHz and the corresponding minimum and maximum when the measurement was repeated 10 times. The corresponding distances were calculated from mean-distance-voltage relationship. Output voltages V_L1_ from the circuit in Fig. 3 are shown (10-time amplified).

Dist. [mm]	Mean V_L1_[mV]	Min. V_L1_[mV]/Dist. [mm]	Max. V_L1_[mV]/Dist. [mm]
0	5.3	5.2/−0.0001	5.3/0.0001
1	18.1	18.1/0.9998	18.1/1.0002
2	38.1	37.1/1.9522	38.4/2.0165
3	57.7	57.7/2.9993	57.8/3.0012
4	81.3	81.3/3.9996	081.3/4.0003
5	102.5	102.1/4.9784	103.1/5.0306
6	119.2	103.2/5.9984	122.4/6.0023
7	141.1	141.0/6.9990	141.1/7.0014
8	155.8	155.3/7.9580	156.1/8.0185
9	167.7	167.7/8.9994	167.7/9.0006
10	179.8	179.8/9.9974	179.9/10.0054
11	189.4	189.0/10.9488	190.3/11.1244
12	197.1	196.8/11.9630	198.1/12.1485
13	204.1	204.0/12.9777	204.3/13.0201
14	209.7	209.4/13.9408	210.2/14.1104
15	214.5	214.3/14.9569	215.0/15.1072

#### Compensation based on adaptation of the input

**Figure 6:**
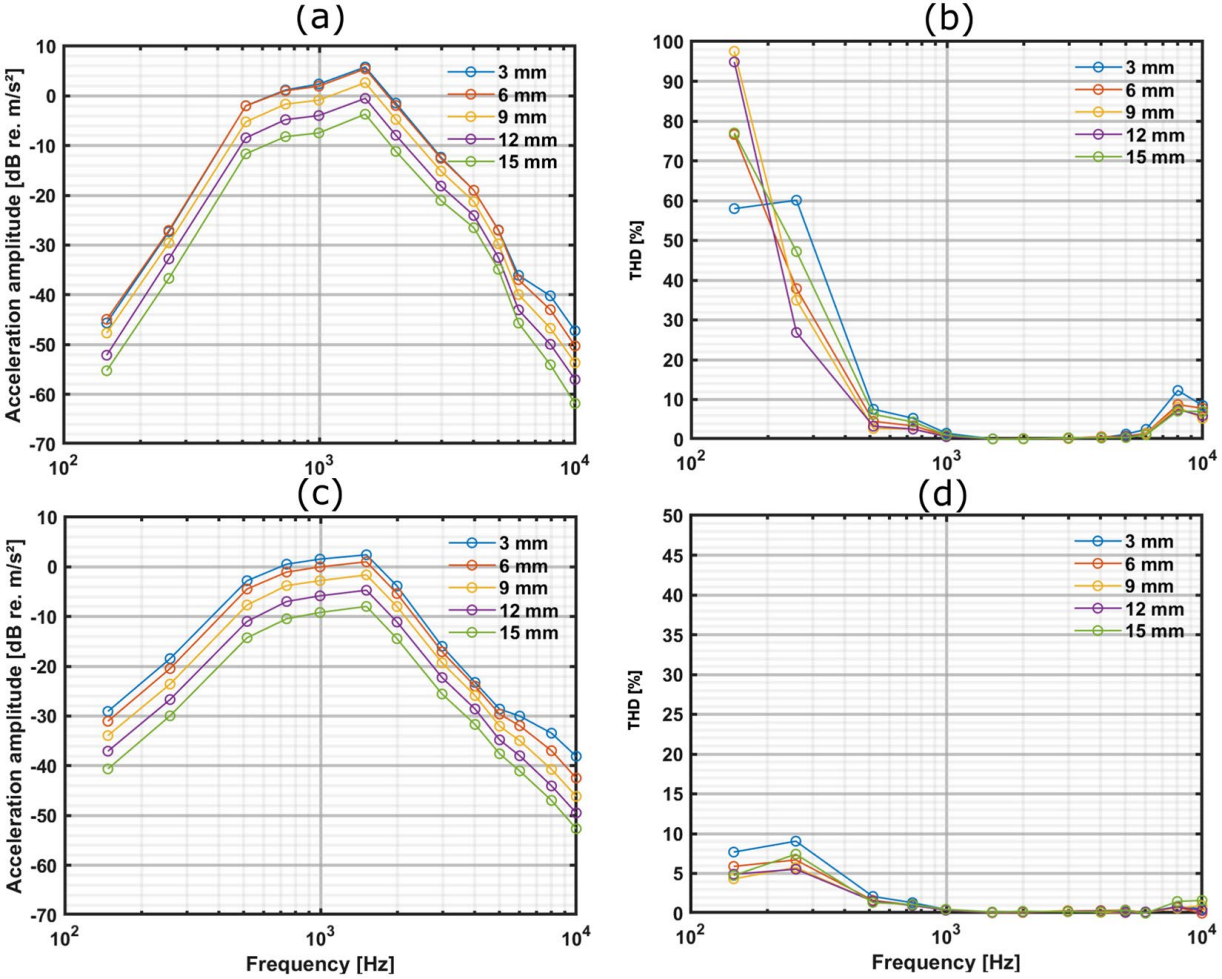
Output of TBCI driven with AP 304/our system without compensation. (**a**) Acceleration amplitude of the TBCI measured on the artificial mastoid driven with the AP 304 at distances from 3 to 15 mm. The input voltage to the AP 304 was 0.1 V_p_. (**b**) Shows the corresponding THD. (**c**) Acceleration amplitude of the TBCI measured on the artificial mastoid driven with our developed transmission system at distances from 3 to 15 mm. The input voltage was A_c_ = 1.5V_p_ with 20% modulation index. (**d**) Shows the corresponding THD. Only measurements at distances of 3, 6, 9, 12 and 15 mm are shown for better visualization.

The unknown distances occurring e.g. in the intraoperative situation were determined by measuring the voltage amplitude V_L1_ at 1 kHz. The amplitudes of measured voltages were compared to the average voltage amplitudes explained in the previous section (Fig. 5). From the measured V_L1_ the distances between the transmitter and the receiver coil were determined using Table 1. At the determined distances, the acceleration amplitude offset from the desired value (at 3 mm, 1 kHz) was determined from the measured amplitude – distance relationship depicted in Fig. 6c. We simply added the measured acceleration amplitude offset to the reference input (A_c_ = 1.5 V_p_/m = 20%) at all determined distances (0–15 mm) using the aforementioned “Stimulation strategy” protocol. This provided a similar BC transducer output vibration amplitude level, independent of distance changes because the transmission circuit behaved mainly in a linear fashion. This simplified compensation strategy was sufficient to adapt the output signal level at all distances and avoid non-linearity to achieve the desired acceleration amplitude with satisfactory accuracy required for the intended intraoperative applications. However, more sophisticated compensation procedures that take frequencies and non-linear input-output dependencies for a broader dynamic range into account will possibly further improve the accuracy of the system. Finally, the constancy of the acceleration output of the BC transducer was verified on the artificial mastoid when driven with our transmission system using the adapted input signal.

## Results

### Evaluation of the transmission system

#### AP 304

The acceleration output amplitude of the TBCI driven with the AP 304 showed (Fig. 6a) an average 11.0 ± 2.6 dB (mean value (MV) ± standard deviation (SD)) drop across frequencies between 0.1 and 10 kHz from the maximum amplitude when the distance between the AP 304 and receiver coil was increased from 0 to 15 mm. However, the average acceleration amplitude drop between 0.1–10 kHz was only 1.4 ± 0.8 dB (MV ± SD) within the distance range that can be expected in implanted patients (5 to 7 mm). In the main frequency range between 1.0 and 6.0 kHz, the THD was below 2.6% at distances between 5 and 7 mm. However, at frequencies below 1.0 kHz and above 8 kHz, higher distortions did occur (Fig. 6b).

#### Our transmission system

The acceleration amplitude of the TBCI when driven with our developed transmission system without adaptation to the distance showed an average drop of 12.0 ± 2.1 dB (MV ± SD) across frequencies between 0.1 and 10 kHz from the maximum amplitude when the distance between the transmitter and receiver coil was increased from 0 to 15 mm (Fig. 6c). Similar to the AP 304, the average amplitude drop was only 2.0 ± 0.5 dB (MV ± SD) in the distance range from 5 to 7 mm. Also, the THD at frequencies between 1.0 and 6.0 kHz was below 0.7% at all distances (Fig. 6d). Although the THD at frequencies below 1.0 kHz was higher at short distances, the THD remained below 12.9% (at 0 mm-not shown in Fig. 6d) at all output levels.

### Distance compensation

#### Amplitude changes

With both the modified AP 304 and our driver system, the maximum output acceleration amplitude of the BC transducer occurred at 6 mm (Fig. 6c) and 3 mm (Fig. 6a), respectively. We used the distance of 3 mm as a reference and calculated the amplitude differences to the maximum level (1.6 dB re. m/s²) at 1 kHz (see Table 2).

**Table 2:** Acceleration amplitude offset with our developed system from the reference distance of 3 mm of the BC transducer with coil–coil distance of our system measured on the artificial mastoid at 1 kHz. The acceleration amplitude differences at 1 kHz between the maximum amplitude at 3 mm (Fig. 6c) and other distances are represented.

Dist. [mm]	0	1	2	3	4	5	6	7	8	9	10	11	12	13	14	15
Δ Amp. [dB]	0.3	0.0	0.0	—	0.8	1.5	2.4	3.5	4.4	5.5	6.5	7.6	8.6	9.7	10.4	11.5

#### Determination of the coil–coil distance

The frequency-dependent voltage across L_1_ in the primary loop is shown in Fig. 5, with distances changed from 0 to 15 mm. There was a distinguishable monotonic increase in V_L1_ at frequencies below 1 kHz when the distance increased between the transmission and receiver coil from 0 to 15 mm (Fig. 5). Furthermore, the *V*_*L*1_ level and the distance dependence remained mainly independent at frequencies below 1 kHz. Therefore, the lower sideband of the FFT amplitude measured across L_1_ with a modulation frequency of 1 kHz was selected to estimate the distance between the transmitter and the receiver coil. To determine the accuracy of the average *V*_*L*1_, measurements were repeated 10 times at each distance to estimate the distance and its variability (Table 1). The maximum variabilities listed in Table 1 indicated that distances were determined within approximately 0.1 mm accuracy when the coil–coil distances were between 0 and 15 mm.

#### Compensation based on adaptation of input

**Figure 7:**
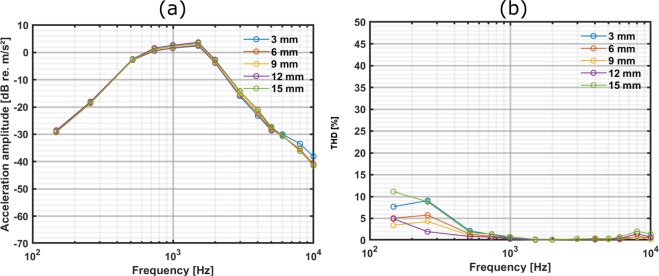
Compensated output of the TBCI using our transmission system. (a) The acceleration amplitude using the adapted modulation input signal to obtain an output independent from distances. (b) Shows the corresponding THD. Only measurements at 3, 6, 9, 12 and 15 mm are shown for better visualization.

The stimulation strategy as described earlier in the “Stimulation strategy” section was used for the adaptation procedure to compensate for transmission losses due to distances (Table 3). Consequently, at distances below 13 mm, the carrier voltages were increased accordingly and for longer distances the modulation index was increased. For compensation, the input modulation signal was adjusted based on the acceleration deviated amplitudes from the maximum level (Fig. 6c) at 3 mm (see Table 2). This simplified protocol, taking neither frequency-specific effects nor non-linearity of A_C_ into account was followed throughout the entire compensation procedure to obtain the results depicted in Fig. 7.

**Table 3:** The adapted modulation signal (A_c_/m) to obtain a similar acceleration amplitude level as compared to 3 mm when the TBCI was driven with our transmission system. In this scenario, a 3-mm distance was chosen as the reference because it provided the maximum acceleration amplitude.

Dist. [mm]	**0**	**1**	**2**	**3**	**4**	**5**	**6**	**7**
Ac[V]/m[%]	1.54/20	1.51/20	1.50/20	1.50/20	1.64/20	1.78/20	1.97/20	2.24/20
Dist. [mm]	**8**	**9**	**10**	**11**	**12**	**13**	**14**	**15**
Ac[V]/m[%]	2.48/20	2.83/20	3.18/20	3.61/20	4.00/20	4.00/23	4.00/24	4.00/28

The acceleration amplitude level of the TBCI when driven with our developed transmission system and adaptation of the input signal using this simple compensation scheme showed an amplitude variation of 2.3 dB (max.– min.) between 0.5 and 6 kHz in the intended range between 0 and 15 mm (Fig. 7a). Moreover, the acceleration amplitude varied only 1.1 dB at 1 kHz where the compensation procedure and adaptation of the input was performed. The THD at frequencies between 1.0 and 6.0 kHz was below 0.8% (Fig. 7b). The THD at frequencies below 1.0 kHz was higher, but remained below 11.1% at all frequencies in the investigated range.

## Discussion

The present study demonstrates the development and evaluation of a stimulation method that provides a system to transmit well-defined stimuli amplitudes to the TBCI. This avoids the use of a (modified) external audio processor to stimulate implantable hearing devices intra- and postoperatively. This study used a simulated scenario to drive the TBCI with AP 304 and measure the acceleration amplitude changes using an artificial mastoid. This provided an average amplitude drop of 11.0 ± 2.6 dB (MV ± SD) between 0.1 and 10 kHz (Fig. 6a) at distances ranging from 0 to 15 mm. Measurements using an artificial mastoid may produce different levels than those seen in patients, as coupling with TBCIs differs from that found with common bone conduction implants integration. However, the relative measurements are still valid. Additionally, the THD measured on an artificial mastoid indicates that the signal is properly transmitted without non-linear distortion in the transmitter. The absolute results in THD determined here may be equally affected compared to the implant screwed to the mastoid bone because of the difference in mechanical point impedances but can be used for relative comparison. Nevertheless, we chose the artificial mastoid as reference for the input impedance because the stimulation site and ways of fixation of transcutaneous devices is less limited making the standardization of the mechanical load, i.e. a skull simulator difficult. In a previous study^10^, the AP 304 was used to stimulate the BC-FMT and we measured the displacement amplitude of the bone close to the implant intraoperatively with LDV and the outer ear canal sound pressure level (OECSPL) with a probe microphone in the auditory ear canal. The goal of this earlier study was to establish an easy affordable intraoperative OECSPL method to determine the functionality of BB implants objectively as an alternative to LDV measurements. There, one of the possible sources of variability in the measured displacement and OECSPL could have been transmission losses due to unknown coil–coil distances. Hence, we decided to develop a transmission system to overcome this unwanted output variability and the limited availability of such modified processors. In addition, the modified AP 304 contains a non-isolated electrical input that requires additional measures for electrical connection in terms of isolation requirements by medical device regulations.

There have been attempts to design a coupling-insensitive inductive link using different types of tuning such as critical coupling^4^ or stagger tuning^5^. These approaches are used to flatten the gain of the transmission line like in the audio processors of transcutaneous devices in a desired distance range. However, these methods are only effective at a limited range. For example, in BB implantation the skin flap thickness above the receiver coil has to remain below 7 mm to assure optimal transmission^3^. In our application, we had no focus on limitation for power consumption and vary the coil–coil distances beyond the normal range expected in patients using the device. Therefore, we decided to use our compensation procedure based on determination of the coil–coil distance and adaptation of the input signal according to the increased distance to provide a more flexible system for larger distances. To estimate the distance, we first attempted to measure the current amplitude flowing into the parallel LC (inductor-capacitor) resonator circuit^6^. However, the current changes due to the distance variation were not as large as the changes of the voltage amplitude across the transmission coil. For this reason, we chose the voltage across the transmitter coil to determine the distances. In the present study, we only considered the distance between the two coils as the source of transmission losses. However, the lateral misalignment and angular displacement of the coils also can cause unwanted transmission losses which have not been investigated. Using the transmission system with the adapted input to drive the BB in a simulated scenario using the artificial mastoid demonstrated a maximum 2.3 dB acceleration amplitude error at frequencies from 0.1 to 6.0 kHz, adequate for most measurement purposes. This was achieved by estimating the distances between the transmitter and receiver coil and applying a simple compensation based on the acceleration amplitude changes at 1 kHz. However, the acceleration amplitude variation was only about 1 dB (Fig. 7a) at 1 kHz using this compensation procedure, which indicates that the procedure can be further refined by extending it for each frequency individually to increase accuracy. Another improvement for better accuracy is to create an acceleration amplitude lookup table in response to different input levels at a certain frequency to compensate for small non-linearity in the modulator amplitude level A_c_. In this case, an interpolation method can be used to determine which input level provides the desired output acceleration level. However, our goal here was to perform the intraoperative functionality test at only one stimulus level. Expanding the fixed level stimulus with our system to a usable dynamic range would require more than a 60 dB range to address electrophysiological or audiometric threshold tests. Our results indicate this to be feasible with the same accuracy demonstrated in this study (see Supplementary Fig. 1 online). However, the modulation signal provided to the amplifier should avoid to exceed the power supply voltage to circumvent higher harmonics caused by the amplifier’s saturation. This can be solved by using more batteries or a medical-grade power supply with higher output voltages such as a power supply module (EN/IEC60601–1, 15 V KMD15–1515, TDK-Lambda Corporation) with ±15 V outputs. Theoretically, this allows for higher output amplitudes for research and testing purposes and is limited only by the implant.

The transmission and measurement system needed to be electrically isolated. To fill this gap our custom-made transmission system was developed with the electrical isolation from the powerline using battery-driven circuitry and an approved power supply (EN/IEC60601-1, KMS40-12, TDK-Lambda Corporation) for the data acquisition system. Although other measures will be necessary for usage under intraoperative conditions, our approach bears the potential to fulfil legal, ethical and technical requirements to perform such tests. The carrier frequency of 151 kHz was preferred to frequencies below 125 kHz (which were the limitations of an earlier isolated DAQ system) because lower total harmonic distortion was obtained measuring the output acceleration amplitude of the BB using the artificial mastoid. Higher harmonics produced by the triangular carrier waves, left to be filtered by the low pass characteristics of the implant circuit, appeared of minor importance for distortion. The THD at frequencies between 1.0 and 6.0 kHz was below 0.8% when the TBCI was driven with our system. However, using a carrier signal with frequency below 125 kHz may have the advantage that it would have been possible to use another electrically isolated USB-driven data acquisition system (NI-USB 6218, National instrument, USA) in our system, making the power supply obsolete.

Our developed system can drive AM modulation-based systems with better precision over an extended distance and dynamic range and can be adapted to medical law constraints. Further, it requires no product or company specific components for which technical specifications are unavailable or only limited accessible. This enables many more experiments that were attempted in the past but suffer from either technical or limitations arising from current medical law requirements. Schnabl *et al*.^11^ applied a new acoustic measurement method using a surface microphone attached to the forehead to assure the integrity of the BB after implantation. In their study, an external audio processor (Amadé, MED-EL, Innsbruck, Austria) was used to stimulate the BB in their measurement setup which had the same limitation for properly-defined input to the device. This study featured a non-calibrated measured surface microphone output, but a more recent study describes an improved surface microphone^12^.

Although our system was originally developed and tested with the BB, the Vibrant Soundbridge and some other implants rely on the same transmission principle. Winter *et al*.^13^ described an intra- and post-operative reverse transfer function (RTF) measurement method in patients implanted with the Vibrant-Soundbridge (VSB, MED-EL, Austria), to determine the coupling efficacy of the floating mass transducer (FMT) using a modified external processor to drive the FMT. In that study, the stapes footplate vibration was intraoperatively recorded as the reference to correlate it to the proposed reverse transfer function method when the FMT was stimulated with the same external processor. In another study by Verhaegen *et al*. using a modified external processor as a stimulus transmission device, auditory steady-state response measurement was used as a feedback tool during the coupling to determine the best FMT position^14^. However, they reported that the limitations of the audio processor prevented higher required levels of stimulation that could be achieved with the system developed here. Another study by Radeloff *et al*.^15^ used the same method of stimulation by applying the sound stimuli to the microphone inlet of a VSB external processor via a silicone tube and measured compound action potential thresholds intraoperatively to assure effective FMT coupling. Colletti *et al*. introduced^16^ an intraoperative electrocochleography to find optimal coupling of the FMT to the round window when the implant was driven by a modified AP404 audio processor. Recently, an optimized VSB -Chirp auditory brainstem response measurement was introduced to intraoperatively determine the aided threshold of the patient and the integrity of the VSB implant. In this study, a Samba audio processor (MED-EL, Innsbruck, Austria) with a wireless streamer was used to present the acoustical stimulus to the implant^17^. Although this approach complies with the electrical isolation requirements, our system adds additional benefit by providing more accurate and higher input over a broader range of stimulation distances that are relevant in intraoperative situations. Providing a well–characterized stimulus has a greater importance in all these studies where the conclusion relies on the comparison among individuals or different studies. All of the previous studies drive the implant using a modified audio processor that is optimized for patients’ daily use; this can potentially cause variability in vibrational, acoustical or electrophysiological measurement results. This can occur because the coil–coil distances or skin thicknesses are larger than normal during surgeries or when there is still swelling or bandages at the surgical area and the distances beyond the intended range cause transmission losses that prohibit adequately-defined input levels.

## Conclusion

In summary, the AM-stimulation with distance compensation described here provides long-range transmission for accurate input to the TBCI in intraoperative applications. Moreover, it can easily be further improved to provide for better accuracy and output than currently available in common sound processors.

## Supplementary information

Supplementary information.

## Data Availability

The data used to support the findings of this study are available from the corresponding author upon request.
