# Supplementary material for: A Precision Driver Device for Intraoperative Stimulation of a Bone Conduction Implant
Source: Sci Rep. 2020 Feb 4;10:1797. doi: 10.1038/s41598-020-58512-7 (PMC7000405; doi:10.1038/s41598-020-58512-7)
Supplement: Supplementary file 1 — Supplementary information. [file 41598_2020_58512_MOESM1_ESM.pdf]

A Precision Driver Device for Intraoperative Stimulation of a Bone Conduction Implant

Mohammad Ghoncheh [1,2], Thomas Lenarz [1,2], Hannes Maier [1,2]

[1] Cluster of Excellence Hearing4all

[2] Department of Otolaryngology and Institute of Audioneurotechnology (VIANNA), Hannover Medical School, Hannover, Germany

Supplementary Material

Corresponding author: Mohammad Ghoncheh

Cluster of Excellence Hearing4all, Medizinische Hochschule Hannover
Klinik für Hals-Nasen-Ohrenheilkunde
Stadtfelddamm 34
30625 Hannover, Germany
Email: Ghoncheh.Mohammad@MH-Hannover.de

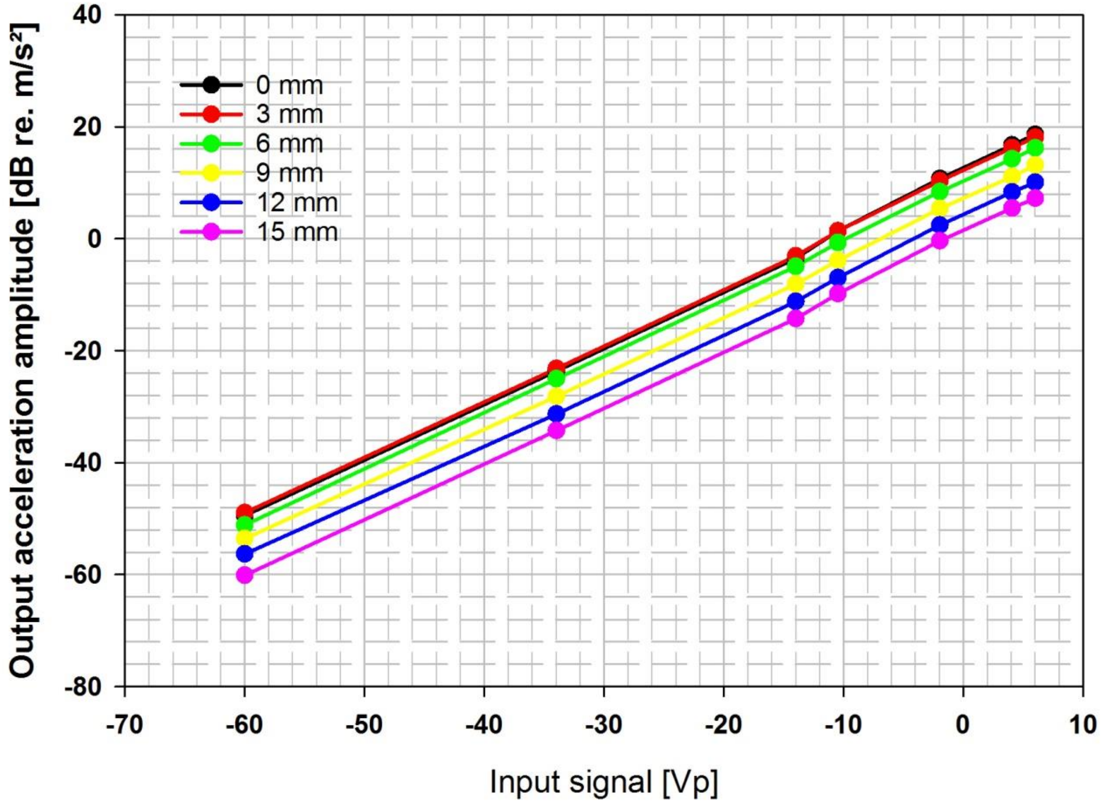

Supplementary figure 1: Example of the linear input-output characteristics of our developed transmission system/protocol measured on the artificial mastoid. The input modulation signal was increased from -60 to 6 dBV at 1 kHz at 0, 3, 6, 9, 12 and 15 mm spacing between the transmitter and receiver coils resulting in a mainly linear relationship between the input voltage and the acceleration output in more than a 60 dB range.
